# Mediterranean diet and remission in rheumatoid arthritis: findings from the EIRA study

**DOI:** 10.1016/j.ero.2026.100201

**Published:** 2026-06-03

**Authors:** Anna-Maria Lampousi, Lars Klareskog, Bénédicte Delcoigne, Helga Westerlind, Lotta Ljung, Johan Askling, Lars Alfredsson, Daniela Di Giuseppe

**Affiliations:** 1 Division of Clinical Epidemiology, Department of Medicine Solna, Karolinska Institutet, Stockholm, Sweden; 2 Division of Rheumatology, Department of Medicine Solna, Karolinska Institutet, Stockholm, Sweden; 3 Stockholm Health Service, Center for Rheumatology, Academic Specialist Center, Stockholm, Sweden; 4 Institute of Environmental Medicine, Karolinska Institutet, Stockholm, Sweden; 5 Center for Occupational and Environmental Medicine, Region Stockholm, Sweden

## Abstract

**Objectives:**

Newly diagnosed patients with rheumatoid arthritis (RA) often ask whether diet can influence disease activity, yet evidence remains limited. We investigated whether prediagnosis adherence to a Mediterranean diet is associated with remission 6 months after diagnosis.

**Methods:**

We included incident RA cases from the Swedish Epidemiological Investigation of Rheumatoid Arthritis study, with follow-up through the Swedish Rheumatology Quality Register. Remission at 6 months was defined using 5 alternative criteria (eg, 28-joint Disease Activity Score [DAS28], Boolean or combinations of their components). Prediagnosis diet was measured with a 124‑item food‑frequency questionnaire, and Mediterranean diet adherence was scored from 0 to 9. Modified Poisson regression estimated adjusted risk ratios (RRs) and 95% CIs for remission, comparing the highest vs the lowest Mediterranean diet tertiles. Analyses were stratified by RA autoantibody status (rheumatoid factor and/or anticitrullinated protein antibodies).

**Results:**

Among 1510 incident RA cases (77% autoantibody-positive), higher Mediterranean diet adherence was associated with DAS28 remission in autoantibody-positive RA (59% vs 49%, RR: 1.24; CI: 1.07-1.44), but not in autoantibody-negative RA (49% vs 58%, RR: 0.84; CI: 0.65-1.09). A similar pattern appeared for Boolean remission (autoantibody-positive: 35% vs 28%, RR: 1.24; CI: 0.98-1.57; autoantibody-negative: 31% vs 32%, RR: 1.00; CI: 0.67-1.49). In contrast, Mediterranean diet adherence was not associated with minimal objective inflammatory activity (28-swollen joint count ≤ 1 and C-reactive protein ≤ 1 mg/dL).

**Conclusions:**

Greater adherence to a Mediterranean diet at RA onset is associated with higher chances of remission in autoantibody-positive, but not in autoantibody‑negative RA.

**WHAT IS ALREADY KNOWN ON THIS TOPIC**

• Current dietary guidelines for patients with rheumatoid arthritis (RA) recommend adhering to a healthy overall dietary pattern, such as the Mediterranean diet.
• These recommendations are largely based on small intervention studies conducted in individuals with long‑standing RA.

**WHAT THIS STUDY ADDS**

• Higher adherence to a Mediterranean diet at RA onset was associated with a greater likelihood of achieving remission 6 months after diagnosis.
• This association was observed only among autoantibody‑positive individuals and was driven primarily by improvements in patient‑reported symptoms rather than objective inflammatory measures.

**HOW THIS STUDY MIGHT AFFECT RESEARCH, PRACTICE OR POLICY**

• The findings support the potential value of incorporating diet into early conversations about patient‑reported well‑being in RA and point toward the need for more personalised approaches to lifestyle management.

Alt-text: Unlabelled box dummy alt text

## INTRODUCTION

Rheumatoid arthritis (RA) is an inflammatory disease with a prevalence of around 0.5% to1% [1]. It is characterised by synovial inflammation leading to joint pain, swelling, and, if left untreated, progressive joint damage and disability [2]. Both environmental/lifestyle and genetic factors contribute to the pathogenesis of the disease [3,4], with major differences between autoantibody-positive and autoantibody-negative RA [5,6].

Current treatment strategies for RA emphasise early and aggressive intervention to achieve clinical remission or low disease activity, with methotrexate (MTX) as first-line treatment in most countries [7]. Besides medication, lifestyle factors such as diet are receiving growing attention, as patients often seek guidance on how they themselves can actively reduce disease activity. Both the European Alliance of Associations for Rheumatology (EULAR) and the American College of Rheumatology (ACR) recommend adherence to a healthy overall diet, rather than focusing on specific food items [8,9]. Among dietary patterns, the ACR specifically recommends a Mediterranean-style diet, recognised for its potential anti-inflammatory properties [10]. Although high adherence to a Mediterranean diet has been linked to a reduced risk of developing (autoantibody-positive) RA [11,12], information regarding its impact on the prognosis of RA remains limited. Although 3 randomised controlled trials (RCTs) and 1 nonrandomised intervention study suggest benefits, they involve populations with long-standing RA, potentially obscuring any effects on disease progression [[13], [14], [15], [16]]. Moreover, evidence is lacking on whether the effect of a Mediterranean dietary pattern on RA prognosis is valid for all patients with RA or is limited to 1 of the 2 major subsets, that is, antibody-positive vs -negative RA.

The aim of this study was therefore to investigate the association between adherence to a Mediterranean dietary pattern and remission at 6 months (overall and separately by objective inflammatory and subjective components) in newly diagnosed RA, and whether this association differs by RA autoantibody status.

## METHODS

### Study population

This study included incident cases of RA from the Swedish Epidemiological Investigation of Rheumatoid Arthritis (EIRA) study, a population-based case-control study previously described in detail [17]. Briefly, since 1996, newly diagnosed adults from central and southern Sweden have been continuously invited to participate, along with a random sample of (matched) controls selected from the national population register. RA diagnoses were made by rheumatologists, and classification of cases was done using either the ACR 1987 criteria or the 2010 ACR/EULAR classification criteria. All participants were asked to complete a questionnaire covering lifestyle and socioeconomic factors and were invited to donate blood.

For the present analysis, we included RA cases who participated in EIRA between November 2005 and March 2018, when a food frequency questionnaire (FFQ) was incorporated into the EIRA questionnaire. The participation rate among cases was 91%. These individuals were evaluated for remission status at 6 months after the RA diagnosis (defined from a visit occurring within a window of 150-269 days), irrespective of their exact treatment during this period. Data on disease activity (to calculate remission status) were retrieved from the Swedish Rheumatology Quality Register (SRQ), which is a digital longitudinal surveillance system that covers 87% of all RA cases in Sweden [18]. The analytical sample consisted of 1510 newly diagnosed RA cases from 2247 EIRA cases enrolled between 2005 and 2018, after exclusions for invalid FFQ data (n = 6), lack of RA diagnosis in the SRQ (n = 51), and missing 6‑month follow-up data (n = 680) (Supplementary Fig S1).

Participants provided informed consent, and ethical approval was obtained from the Regional Ethical Review Board at Karolinska Institutet, Stockholm, Sweden (DNR 2021-00125).

### Outcome assessment

Information on disease activity, including 28-swollen joint count (SJC), 28-tender joint count (TJC), C-reactive protein (CRP), pain assessed using a visual analogue scale (VAS), patient global assessment (PGA) on the VAS scale, the 28-joint Disease Activity Score (DAS28), and DAS28-CRP, was collected at the 6-month follow-up visit and obtained through SRQ. Remission was assessed based on DAS28 (DAS28 < 2.6 or DAS28-CRP < 2.4) or Boolean criteria (SJC ≤ 1, TJC ≤ 1, PGA ≤ 20, and CRP ≤ 1 mg/dL) [19]. The remission criteria were complemented with 2 combinations of included components reflecting minimal subjective symptoms (TJC ≤ 1 and PGA ≤ 20) and minimal objective inflammatory activity (SJC ≤ 1 and CRP ≤ 1 mg/dL), as well as an outcome based on reported pain (VAS pain ≤ 10 mm).

### Diet and covariate assessment

Habitual diet for the year prior to RA diagnosis was assessed with a 124-item FFQ validated in a Swedish population [20]. Participants were asked how often they consumed each food item, with preassigned responses ranging from ‘0 times/mo’ to ‘≥ 3 times/d’, except for certain drinks (ie, milk, sweetened beverages, coffee, and tea), sugar, yoghurt, cheese, and bread, for which participants could indicate the exact frequencies. Alcoholic beverages included beer, wine, and spirits. The quantity in centilitres of each type of beverage consumed per occasion was assessed as an open-ended question. Average alcohol consumption in g/d was calculated based on the frequency, type, and quantity of alcoholic drinks. Energy (kcal/d) and lipid intakes were calculated by multiplying the frequency of consumption of each item by its energy and lipid content, respectively, based on age- and sex-specific portion sizes from the Swedish National Food Agency database. The regular use of dietary supplements, including multivitamins (with or without minerals), for at least 3 months over the past 5 years was also assessed through the questionnaire.

Adherence to a Mediterranean dietary pattern was assessed using the Mediterranean diet score, which ranges from 0 to 9, with higher scores indicating greater adherence [21]. One point was assigned for each of the following dietary components: intake above the sex-specific study population median for vegetables, legumes, fruits and nuts, cereals, fish, and the ratio of monounsaturated to saturated fatty acids; intake below the sex-specific median for meat and meat products, and full-fat dairy; and moderate alcohol consumption, defined as 10 to 50 g per day for men and 5 to 25 g per day for women.

Information on other lifestyle factors up to RA diagnosis, including smoking and physical activity, as well as age, sex, education, parity, weight, and height, was collected through the EIRA questionnaire. Female participants were additionally asked about age at menarche, and use of oral contraceptives and hormone replacement therapy.

### Biochemical analyses

Autoantibody positivity was defined as being positive for either rheumatoid factor (RF) and/or anticitrullinated protein antibodies (ACPAs). RF positivity was assessed by routine methods at each clinical unit. ACPA were measured at Karolinska Institutet using blood samples collected at diagnosis by the clinic. ACPA levels were determined as anti-cyclic citrullinated peptide 2 (anti-CCP2) IgG using the Immunoscan-RA MARK 2 ELISA test (Euro Diagnostica AB) [22], and ACPA positivity was defined as anti-CCP2 concentrations ≥25 AU/mL. High autoantibody levels were defined as having either RF or ACPA levels exceeding 3 times their respective positivity cut‑off values (RF ≥15 IU/mL and/or ACPA ≥75 AU/mL). Blood samples were genotyped according to previously described methods [23]. Individuals carrying any of the HLA–DRB1*01, HLA–DRB1*04, or HLA–DRB1*10 alleles were classified as having a shared epitope (SE) allele.

### Statistical analyses

Baseline characteristics of study participants and individuals lost to follow-up were reported as means with standard deviations for continuous variables and as proportions (%) for categorical variables. Risk ratios (RRs) with 95% CIs for remission at 6 months after diagnosis were estimated using modified Poisson regression models in relation to Mediterranean diet score tertiles, which were defined based on all participants. The resulting tertiles corresponded to a commonly used classification of Mediterranean diet adherence, defining low (0-3), medium (4-5), and high (6-9) adherence [24]. Accordingly, the lowest tertile, reflecting low adherence, was used as the reference category. These analyses were stratified by RA autoantibody status and by level of autoantibodies, as well as sex, presence of the SE allele, and smoking status. We also assessed interactions on the multiplicative scale between these factors and Mediterranean diet score tertiles by including product terms in the models. Separate analyses were performed for female smokers who were both RF and ACPA positive, factors linked to typical autoantibody-positive RA. To assess potential effect modification by MTX use, we conducted a sensitivity analysis restricted to individuals who initiated MTX monotherapy within the first 3 months after diagnosis. Those who discontinued MTX during follow‑up (n = 92) were excluded, resulting in an analytical sample of 1043 individuals. The Poisson regression model 1 was adjusted for age, sex, and healthcare region (Stockholm-Gotland, Southeast, West, South, and Uppsala-Örebro). Model 2 included additional adjustments for smoking status (never, former, or current smoker), leisure‑time physical activity (sedentary or any exercise), educational level (<10 years, 10-12 years, or >12 years), body mass index (underweight: <18.5 kg/m²; normal weight: 18.5-24.9 kg/m²; overweight: 25-29.9 kg/m²; and obese: ≥30 kg/m²), total energy intake (sex-specific quartiles), supplement use (yes/no), and cortisone use (yes/no). Model 3 was further adjusted for baseline disease activity: SJC, TJC, PGA, and CRP. For analyses restricted to females, further adjustments were made for parity (quartiles), age at menarche (quartiles), use of oral contraceptives (yes/no), and hormone replacement therapy (yes/no). Missing categorical covariates were handled through the creation of separate indicator categories. Unless otherwise specified, results refer to model 2, as 12% of participants had missing baseline disease activity data used in model 3.

To evaluate the relative contribution of each component of the Mediterranean diet score, we performed leave-one-out analyses. In each model, individual dietary components were sequentially omitted from the score, and associations with remission outcomes were then re-examined by comparing participants in the highest vs lowest Mediterranean diet score tertiles.

Statistical analyses were performed with Stata Statistical Software Release 18 (StataCorp).

## RESULTS

A total of 1510 incident RA cases (77% autoantibody-positive), with a mean age of 54.9 (SD 13.7) years, were included in the analyses. Baseline characteristics of the study participants are summarised in Table 1. Participants with the highest Mediterranean diet score were more likely to be female, have a higher educational attainment, and a generally healthier lifestyle, characterised by a lower prevalence of smoking and a higher prevalence of physical activity, than those with the lowest Mediterranean diet score. Moreover, they had lower CRP levels at baseline and were less likely to carry an SE allele and to be autoantibody-positive. They also scored higher on all individual Mediterranean diet score components, except for the fatty acid ratio, which was similar across groups (Supplementary Table S1). The characteristics of individuals lost to follow-up were comparable with those of study participants included in the analyses, except that they were more likely to come from regions outside Stockholm-Gotland (Supplementary Table S2).

**Table 1:** Characteristics of study participants at the time of RA diagnosis (N = 1510)

Characteristic given as n (%) or mean (SD)	Tertile 1 (0-3)	Tertile 2 (4-5)	Tertile 3 (6-9)
	n = 604	n = 554	n = 352
Age, y	54.6 (14.8)	54.9 (13.6)	55.3 (11.9)
Female participants	400 (66)	399 (72)	267 (76)
Healthcare region			
Stockholm-Gotland	410 (68)	395 (71)	253 (72)
Southeast	39 (6)	41 (8)	28 (8)
West	11 (2)	13 (2)	7 (2)
South	127 (21)	83 (15)	54 (15)
Uppsala-Örebro	17 (3)	22 (4)	10 (3)
Smoking status			
Never smokers	213 (35)	204 (37)	138 (39)
Former smokers	176 (29)	202 (37)	125 (36)
Current smokers	208 (35)	141 (26)	87 (25)
Physically active	472 (79)	452 (82)	309 (88)
High education	282 (47)	317 (57)	232 (66)
Autoantibody-positive	477 (80)	424 (77)	245 (70)
Shared epitope	296 (79)	278 (74)	209 (72)
MTX use	447 (74)	409 (74)	258 (73)
Cortisone use	310 (51)	295 (53)	183 (52)
Body mass index, kg/m^2^	26.0 (4.8)	25.7 (4.6)	25.2 (4.3)
Baseline disease activity			
28-swollen joint count	8.5 (5.8)	7.8 (5.4)	9.0 (6.1)
28-tender joint count	7.9 (6.0)	7.5 (5.5)	8.2 (6.3)
28-joint Disease Activity Score	5.1 (1.4)	4.9 (1.3)	5.0 (1.3)
Patient global assessment, VAS: 0-100 mm	47.2 (25.1)	47.5 (25.8)	46.5 (24.4)
Pain, VAS: 0-100 mm	52.1 (25.7)	50.8 (26.1)	49.4 (24.4)
C-reactive protein, mg/dL	2.1 (3.1)	1.9 (2.8)	1.7 (2.6)

MTX, methotrexate; RA, rheumatoid arthritis; VAS, visual analogue scale.

Missing data (% of total N = 1510) were: smoking status 1.1%, physical activity 0.4%, autoantibody status 0.8%, shared epitope 30.9%, and body mass index 0.3%. For disease activity measures, missingness ranged from 6.7% to 12.9%.

### Mediterranean diet and remission in RA

Most participants achieved minimal objective inflammatory activity (60%), followed by DAS28 remission (52%), minimal subjective symptoms (38%), minimal pain (34%), and Boolean remission (30%). Men were more likely than women to achieve favourable outcomes across all definitions, while outcomes did not differ by autoantibody-positivity or smoking status (Supplementary Table S3).

Individuals in the highest tertile of the Mediterranean diet score had higher chances of achieving favourable outcomes than those in the middle and lowest tertiles across all definitions, except for minimal objective inflammatory activity (Table 2). The corresponding age- sex-, and region-adjusted RRs for the highest vs lowest tertile were: DAS28 remission, 1.15 (95% CI: 1.02-1.31, *P*-trend = .032); Boolean remission, 1.26 (95% CI: 1.03-1.54, *P*-trend = .025); minimal subjective symptoms, 1.25 (95% CI: 1.06-1.47, *P*-trend = .011); minimal pain, 1.14 (95% CI: 0.96-1.37, *P*-trend = .154); and minimal objective inflammatory activity, 1.00 (95% CI: 0.89-1.11, *P*-trend = .969). The RRs remained largely consistent across the fully adjusted models and analyses restricted to individuals on MTX monotherapy, although the CIs became wider and included the null for all outcomes except minimal subjective symptoms (Table 2; Supplementary Table S4).

**Table 2:** Risk ratios and 95% confidence intervals for remission at 6 months postdiagnosis, across tertiles of Mediterranean diet score (N = 1510)

	N total	N remission (%)	RR (95% CI)^a^	RR (95% CI)^b^	RR (95% CI)^c^^,^^d^
DAS28 remission					
Tertile 1 (0-3)	575	290 (50)	Reference	Reference	Reference
Tertile 2 (4-5)	532	274 (52)	1.04 (0.93-1.17)	1.03 (0.92-1.16)	1.05 (0.93-1.19)
Tertile 3 (6-9)	336	188 (56)	1.15 (1.02-1.31)	1.12 (0.99-1.27)	1.14 (1.00-1.30)
			*P*-trend .032	*P*-trend .094	*P*-trend .051
Boolean remission					
Tertile 1 (0-3)	568	161 (28)	Reference	Reference	Reference
Tertile 2 (4-5)	527	159 (30)	1.09 (0.91-1.31)	1.05 (0.87-1.26)	1.08 (0.89-1.30)
Tertile 3 (6-9)	335	114 (34)	1.26 (1.03-1.54)	1.19 (0.97-1.45)	1.25 (1.02-1.53)
			*P*-trend .025	*P*-trend .104	*P*-trend .033
Minimal subjective symptoms					
Tertile 1 (0-3)	580	204 (35)	Reference	Reference	Reference
Tertile 2 (4-5)	534	200 (37)	1.09 (0.93-1.27)	1.07 (0.92-1.25)	1.09 (0.93-1.28)
Tertile 3 (6-9)	342	144 (42)	1.25 (1.06-1.47)	1.22 (1.03-1.44)	1.27 (1.07-1.50)
			*P*-trend .011	*P*-trend .025	*P*-trend .007
Minimal pain					
Tertile 1 (0-3)	580	190 (33)	Reference	Reference	Reference
Tertile 2 (4-5)	535	181 (34)	1.05 (0.89-1.23)	1.03 (0.87-1.20)	1.05 (0.89-1.25)
Tertile 3 (6-9)	345	125 (36)	1.14 (0.96-1.37)	1.10 (0.91-1.32)	1.09 (0.90-1.32)
			*P*-trend .154	*P*-trend .336	*P*-trend .349
Minimal objective inflammatory activity					
Tertile 1 (0-3)	582	349 (60)	Reference	Reference	Reference
Tertile 2 (4-5)	534	325 (61)	1.03 (0.94-1.13)	1.02 (0.92-1.12)	1.01 (0.92-1.12)
Tertile 3 (6-9)	339	198 (58)	1.00 (0.89-1.11)	0.97 (0.86-1.08)	0.96 (0.86-1.09)
			*P*-trend .969	*P*-trend .621	*P*-trend .614

DAS28, 28-joint Disease Activity Score; RR, risk ratio.

Missing data (% of total N=1510) were: DAS28 remission 4.4%, Boolean remission 5.3%, minimal subjective symptoms 3.6%, minimal pain 3.3%, and minimal objective inflammatory activity 3.6%.

a Adjusted for age, sex, and region.

b Adjusted for age, sex, region, smoking status, physical activity, educational level, body mass index, total energy intake, supplement use, and cortisone use.

c Adjusted for age, sex, region, smoking status, physical activity, educational level, body mass index, total energy intake, supplement use, cortisone use, baseline 28-swollen joint count, 28-tender joint count, patient global assessment, and C-reactive protein.

d 1334 participants were included in this analysis.

Stratified analyses by autoantibody status indicated that the association between a higher Mediterranean diet score and a greater likelihood of remission was evident only among individuals with autoantibody-positive RA (Fig). In this subgroup, greater adherence to a Mediterranean diet was associated with higher chances of DAS28 remission (RR: 1.24, 95% CI: 1.07-1.44) and minimal subjective symptoms (RR: 1.24, 95% CI: 1.02-1.51). A similar, though nonstatistically significant, association was observed for Boolean remission (Fig). Effect estimates were comparable across strata of high and low autoantibody levels (Supplementary Fig S2). Notably, the observed associations were also confined to women and carriers of the SE allele, and were most pronounced among female smokers who were both RF and ACPA positive (Supplementary Fig S2). The corresponding RRs were 1.54 (95% CI: 1.09-2.17) for DAS28 remission and 1.62 (95% CI: 1.02-2.58) for minimal subjective symptoms. By contrast, among individuals without the SE allele, those in the highest Mediterranean diet score tertile were less likely to achieve DAS28 remission than those in the lowest tertile. Evidence of multiplicative interaction was observed between Mediterranean diet score tertiles and autoantibody positivity (*P* = .006) and SE carriage (*P* = .001) for DAS28 remission, but not for other outcomes.

**Figure:**
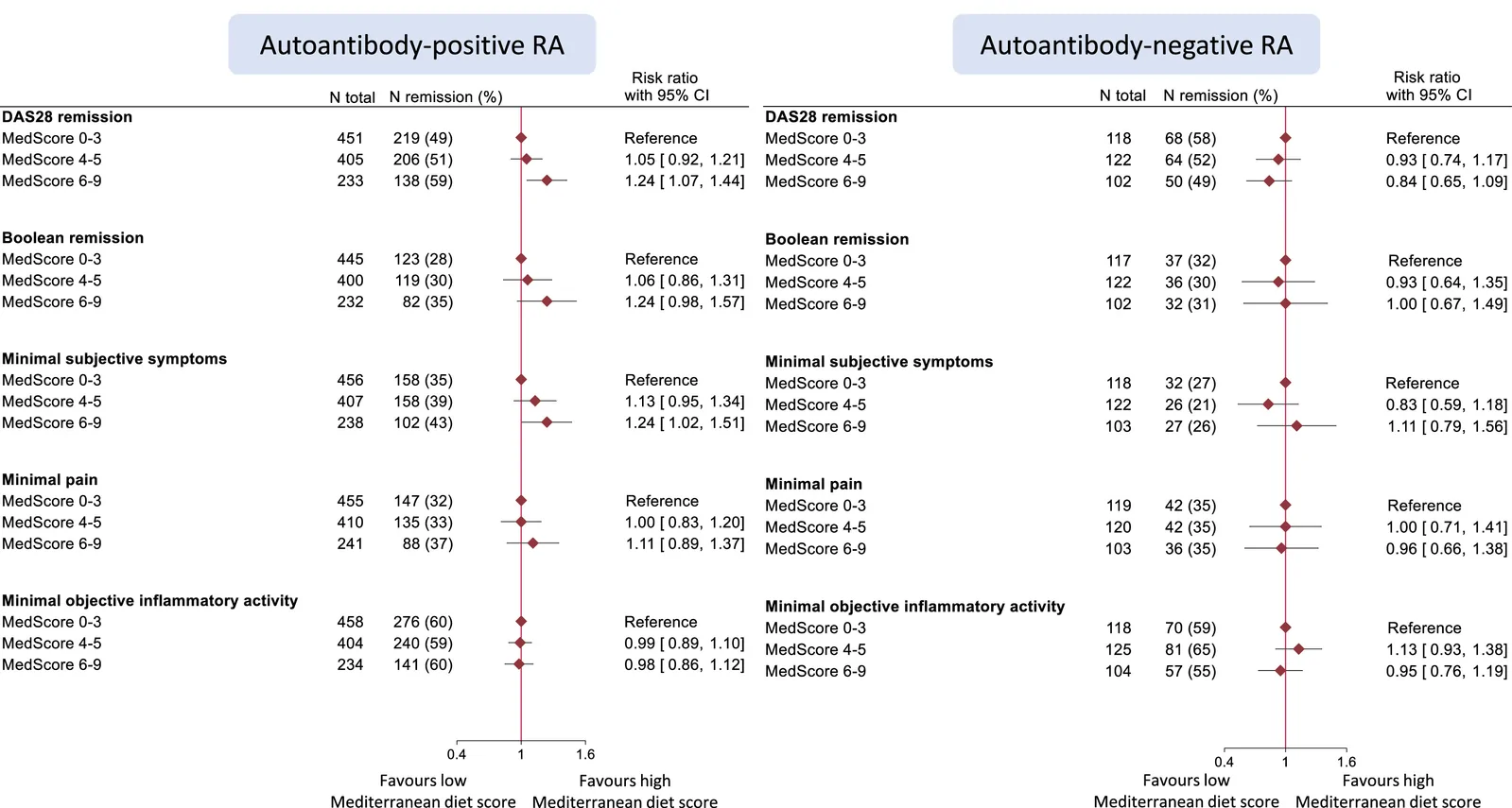
Risk ratios and 95% CIs for remission at 6 months postdiagnosis, across tertiles of Mediterranean diet score, stratified by autoantibody-positive and autoantibody-negative rheumatoid arthritis. Analyses were adjusted for age, sex, region, smoking status, physical activity, educational level, body mass index, total energy intake, supplement use, and cortisone use. DAS28, 28-joint Disease Activity Score; RA, rheumatoid arthritis.

Leave-one-out analyses revealed that excluding the fruit and nuts component from the Mediterranean diet score yielded stronger associations between a higher Mediterranean diet score and DAS28 remission (RR: 1.18; CI: 1.02-1.37), minimal subjective symptoms (RR: 1.28; CI: 1.06-1.54), and minimal pain (RR: 1.21; CI: 0.98-1.50). For Boolean remission, an association was no longer observed when the fish (RR: 1.04; CI: 0.82-1.31) and dairy (RR: 1.05; CI: 0.83-1.33) components were excluded (Supplementary Fig S3). These patterns suggest that the fruit and nuts component may contribute less to the observed associations, whereas fish and dairy may have a stronger influence. Similar patterns were even more evident when analyses were restricted to autoantibody‑positive individuals (Supplementary Fig S4). In the autoantibody‑negative RA subgroup, the absence of an association between a higher Mediterranean diet score and remission outcomes persisted across all leave‑one‑out analyses (Supplementary Fig S5).

## DISCUSSION

We found that patients with RA with greater adherence to a Mediterranean dietary pattern at disease onset had a higher likelihood of remission 6 months after diagnosis. Notably, these associations were observed only among individuals with autoantibody-positive RA and were primarily related to improvements in subjective symptoms rather than objective markers of inflammation.

The potential of a Mediterranean dietary pattern to reduce disease activity in RA has been investigated in 3 RCTs and 1 nonrandomised intervention study, primarily in female patients with established RA, whereas prospective observational studies are notably lacking. One RCT from Sweden (2003) and 1 nonrandomised trial from Scotland (2007), including 51 and 130 patients with RA respectively, reported improved physical function and reduced pain with a Mediterranean-style diet compared with a Western diet or standard dietary advice [13,14]. A more recent RCT from Mexico (2022) involving 106 women with RA found greater benefits when a Mediterranean-style diet was combined with exercise, compared with either intervention alone [15]. Finally, an RCT conducted in Ireland (2024) compared a telehealth-delivered Mediterranean diet with the Irish Healthy Eating guidelines in 44 individuals with RA and reported similar improvements in both groups [16]. Importantly, these studies focused on individuals with established RA, with mean disease durations exceeding 8 years. Our study adds to this evidence by demonstrating an association between higher adherence to a Mediterranean diet and the chance of remission in newly diagnosed patients. Although the assessment of the Mediterranean diet was done at baseline, it is unlikely that the dietary pattern changed within the first 6 months after diagnosis, as shown in a previous publication in Swedish women with RA [25]. However, improvements mainly appeared in patient-reported outcomes rather than in objective inflammatory markers. Previous studies have yielded mixed results regarding the effects of a Mediterranean-style diet on inflammation in RA; 1 RCT reported reductions in CRP levels and SJCs, whereas a nonrandomised trial found no improvements in these measures [13,14]. One possible explanation for the lack of an association between higher adherence to a Mediterranean diet and minimal objective inflammatory activity in our study is that most participants were receiving MTX therapy, which likely contributed to reduced inflammatory activity in both the high‑ and low‑adherence groups. Consequently, associations between dietary adherence and inflammatory activity may have been difficult to detect. In contrast, associations with patient‑reported outcomes may reflect dietary influences occurring in addition to ongoing pharmacologic treatment. Moreover, our finding that higher self-reported adherence to a Mediterranean diet is associated with better chances of remission in autoantibody-positive individuals, particularly female smokers, but not autoantibody-negative individuals, might be explained by modulation of RA-associated autoimmunity. This result is consistent with earlier reports from the EIRA study, which found that higher adherence to a Mediterranean diet was associated with a reduced risk of developing autoantibody-positive, but not autoantibody-negative RA [11]. In line with the mucosal origins hypothesis [26], RA‑associated autoantibodies are thought to arise at mucosal sites, including the intestinal tract, where immune responses may be shaped by diet‑dependent alterations in the microbiota, thereby preferentially influencing autoantibody‑mediated disease pathways [27]. Consistent with this, RA has been associated with gut dysbiosis, characterised by reduced levels of beneficial bacteria such as *Bifidobacterium* and an increased abundance of *Prevotella copri*, particularly in early disease [28].

Biological evidence supports the notion that a Mediterranean diet may help promote remission in RA. This dietary pattern emphasises plant-based foods, fish, and olive oil, while limiting red and processed meats [29]. As a result, it provides a lipid profile rich in monounsaturated and omega-3 polyunsaturated fatty acids, as well as high levels of antioxidants and fibre, factors known to modulate inflammatory pathways and potentially influence autoimmune processes. Compared with conventional Western diets, the Mediterranean diet typically achieves a more favourable omega-6 to omega-3 ratio (2:1 to 4:1 vs 10:1 to 20:1), which has been associated with reduced inflammation [30]. Omega-6 fatty acids, mainly found in vegetable oils, are converted into arachidonic acid in the body, a precursor of proinflammatory eicosanoids. In contrast, omega-3 fatty acids, particularly eicosapentaenoic acid and docosahexaenoic acid found in fish, compete with arachidonic acid for incorporation into cell membranes, reducing the production of proinflammatory eicosanoids [31]. In addition, omega-3 fatty acids can be further metabolised into specialised proresolving lipid mediators, actively promoting inflammation resolution [32]. Importantly, these pathways may also be relevant to immune regulation in autoimmune disease. By altering cell membrane composition, signalling thresholds, and intercellular communication, omega‑3 fatty acids influence the activation and coordination of adaptive immune responses [33]. The Mediterranean diet is also rich in dietary fibre, which influences gut microbiota composition and function through the production of short‑chain fatty acids with hypothesised immunomodulatory properties [34]. Supporting this concept, a feasibility study administering high‑fibre bars or cereals to 36 patients with RA for 28 days demonstrated an increase in circulating regulatory T cells, an improvement in the T helper 1/T helper 17 cell balance, and a reduction in biomarkers associated with bone erosion, alongside improvements in patient‑reported outcomes [27]. These findings are consistent with emerging evidence linking intestinal immune modulation to joint inflammation, potentially mediated by the migration of gut‑derived immune cells to synovial tissues [35]. Another characteristic of the Mediterranean diet is moderate alcohol consumption, particularly red wine, which has been shown to influence inflammatory pathways and adaptive immune responses, including T‑cell regulation [36]. Consistent with this, data from EIRA and other observational studies have reported lower disease activity among alcohol consumers than among abstainers [37,38]. Antioxidants represent another component of the Mediterranean diet that may contribute to improved RA outcomes. Found in fruits, vegetables, olive oil, and red wine, compounds such as vitamins C and E and polyphenols help neutralise reactive oxygen species, which can trigger inflammatory signalling [39]. Moreover, evidence from other autoimmune conditions, such as diabetes, suggests that antioxidant vitamins may reduce disease risk, particularly in forms with stronger autoimmune components [40]. In addition, a recent cross‑sectional study suggested inverse associations between olive oil consumption and RA disease activity, particularly among autoantibody‑positive patients [41].

### Strengths and limitations

This study has several strengths. Its observational design and inclusion of newly diagnosed individuals with RA enabled us to examine the role of a Mediterranean diet in early disease remission, using a larger sample size than typically feasible in intervention studies. The richness of the EIRA data allowed us to explore whether associations were modified by autoantibody-positivity status and other clinical factors, while adjusting for a wide range of potential confounders. Moreover, we had access to detailed disease activity measures through the SRQ.

This study also has some limitations. Dietary intake was assessed using an FFQ, which, although validated and designed to capture habitual intake of commonly consumed foods in Sweden, relies on participants’ memory and their ability to accurately estimate usual intake, and may not fully capture all dietary components. Consequently, some degree of measurement error is likely. However, because diet was assessed before outcome ascertainment, any misclassification is likely to be nondifferential and may have attenuated the observed associations toward the null. In addition, the FFQ captured dietary habits during the year before diagnosis, which may not fully reflect dietary behaviour at the time of diagnosis nor during the following months. Nevertheless, previous research in Sweden has shown that individuals newly diagnosed with RA do not typically make substantial dietary changes following diagnosis [25]. It is more likely that participants with the lowest adherence to the Mediterranean diet, which is an indicator of an overall healthy dietary pattern, may have increased their adherence following diagnosis, potentially leading to attenuated associations. Another limitation is that the Mediterranean diet score reflects relative intake levels within a population, based on median cut-offs, rather than absolute adherence to a traditional Mediterranean diet. Alcohol intake was the only component with a predefined threshold. Consequently, individuals with higher scores do not necessarily follow a traditional Mediterranean diet. Although the absolute intakes of most individual food groups among participants in the highest tertile of the Mediterranean diet score were broadly consistent with a traditional Mediterranean diet, intakes of red and processed meat exceeded recommended levels [29]. Therefore, our findings may not be generalisable to populations with different dietary patterns. Furthermore, although the Mediterranean diet score serves as an indicator of overall dietary quality, it does not capture certain dietary exposures that have been implicated in RA, such as salt intake and consumption of nonalcoholic beverages [42,43]. Future studies examining dietary patterns that encompass a broader range of dietary components are needed to examine whether these exposures independently or jointly influence RA prognosis. In addition, a substantial proportion of EIRA participants lacked SRQ data 6 months after diagnosis. However, this appears unrelated to their Mediterranean diet scores, suggesting a low risk of selection bias. Finally, although we adjusted for a wide range of potential confounders, the possibility of residual confounding cannot be ruled out, particularly given the complexity of dietary and lifestyle behaviours and the absence of detailed information on some factors, such as comorbidities.

### Conclusion

Our findings suggest that greater adherence to a Mediterranean diet may improve short-term prognosis in patients with newly diagnosed RA. The associations were confined to autoantibody‑positive RA, but not autoantibody‑negative RA, and were related mainly to subjective symptoms rather than objective inflammatory measures.

## Editor disclosure

The peer review process did not involve Editorial Board Member Lars Klareskog, and the editorial decision-making was led by editors who were not involved in the creation of this manuscript

## Competing interests

All authors declare they have no competing interests.

## Appendix Supplementary materials

Image, application 1

## Footnotes

Supplementary material associated with this article can be found in the online version at doi:10.1016/j.ero.2026.100201.
