# Supplementary material for: Mediterranean diet and remission in rheumatoid arthritis: findings from the EIRA study
Source: EULAR Rheumatol Open. 2026 Jun 3;2(3):100201. doi: 10.1016/j.ero.2026.100201 (PMC13650335; doi:10.1016/j.ero.2026.100201)
Supplement: Supplementary file 1 [file mmc1.pdf]

Supplementary Material

Mediterranean diet and remission in rheumatoid arthritis: findings from the EIRA study

Anna-Maria Lampousi, Lars Klareskog, Bénédicte Delcoigne, Helga Westerlind, Lotta Ljung, Johan Askling, Lars Alfredsson, Daniela Di Giuseppe

Corresponding author: Anna-Maria Lampousi
Email: annamaria.lampousi@ki.se

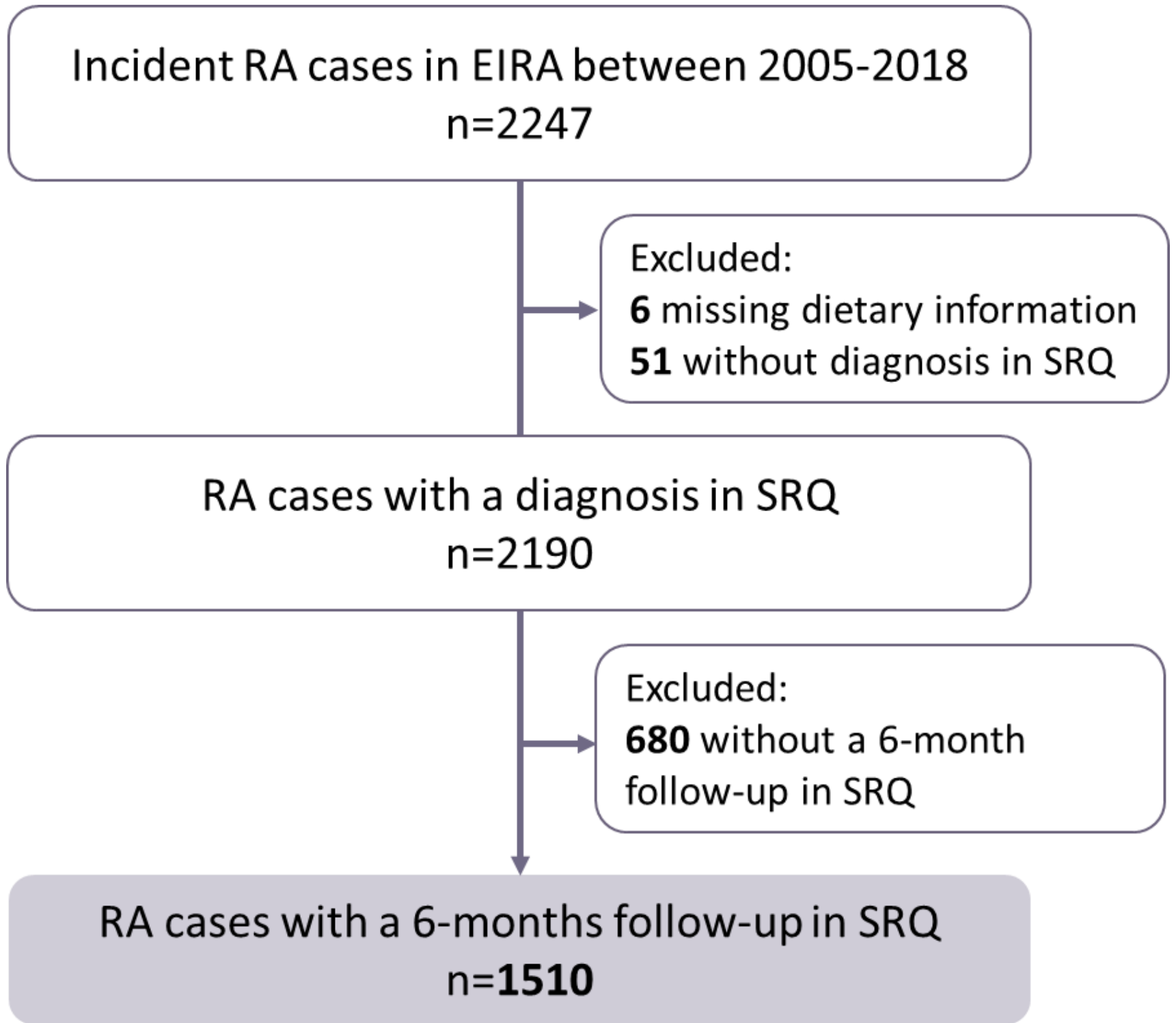

Supplementary Figure 1. Flow diagram of study participants. EIRA, Epidemiological Investigation of Rheumatoid Arthritis; FFQ, Food frequency questionnaire; RA, rheumatoid arthritis; SRQ, Swedish Rheumatology Quality Register

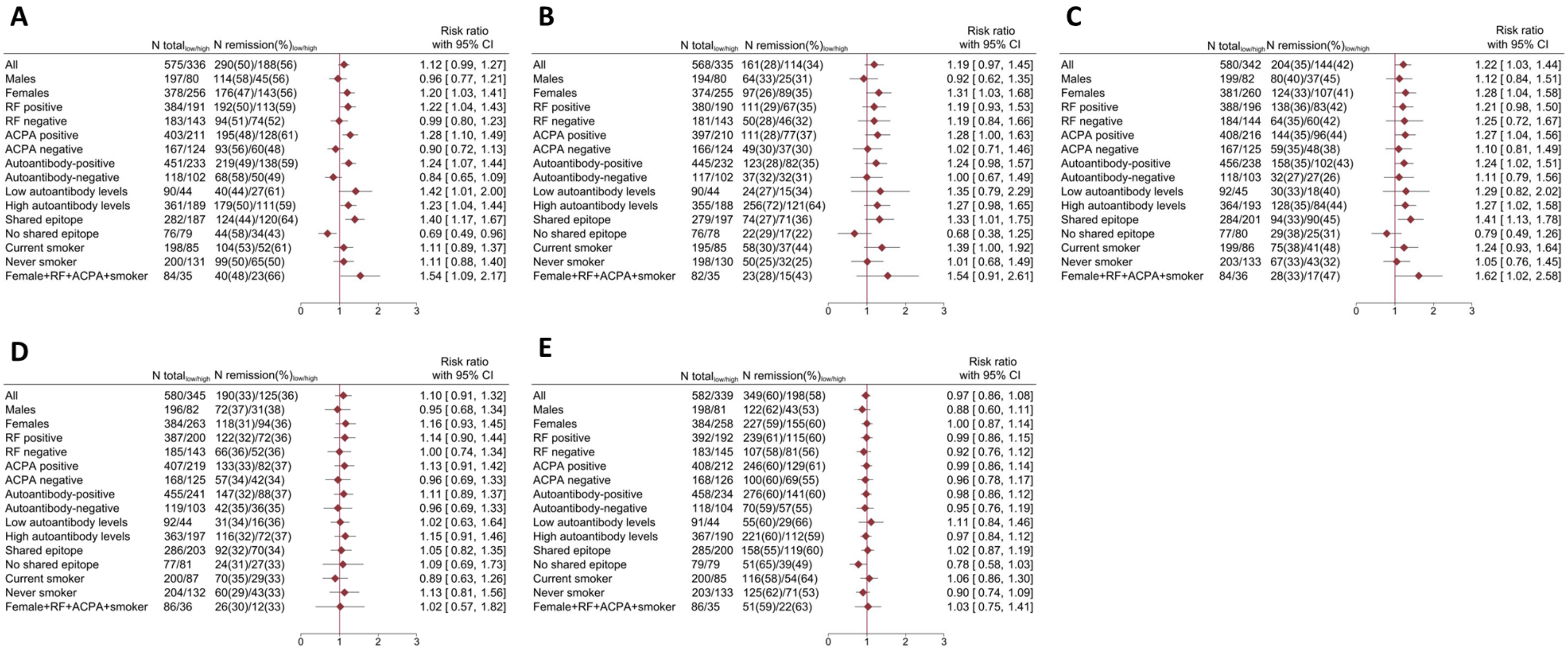

Supplementary Figure 2. Risk ratios and 95% confidence intervals for DAS28 remission (A), Boolean remission (B), Minimal subjective symptoms (C), Minimal pain (D), and Minimal objective inflammatory activity (E) at 6 months post-diagnosis, comparing the highest versus the lowest tertile of Mediterranean diet score. Analyses are stratified by sex, autoantibody-positivity (RF, ACPA, or either), shared epitope status, and smoking status. All models are adjusted for age, sex, region, smoking status, physical activity, educational level, body mass index, total energy intake, supplement use, and cortisone use. Female-specific models include additional adjustments for parity, age at menarche, and use of oral contraceptives and hormone replacement therapy.
ACPA, anti-citrullinated protein antibodies; RF, rheumatoid factor

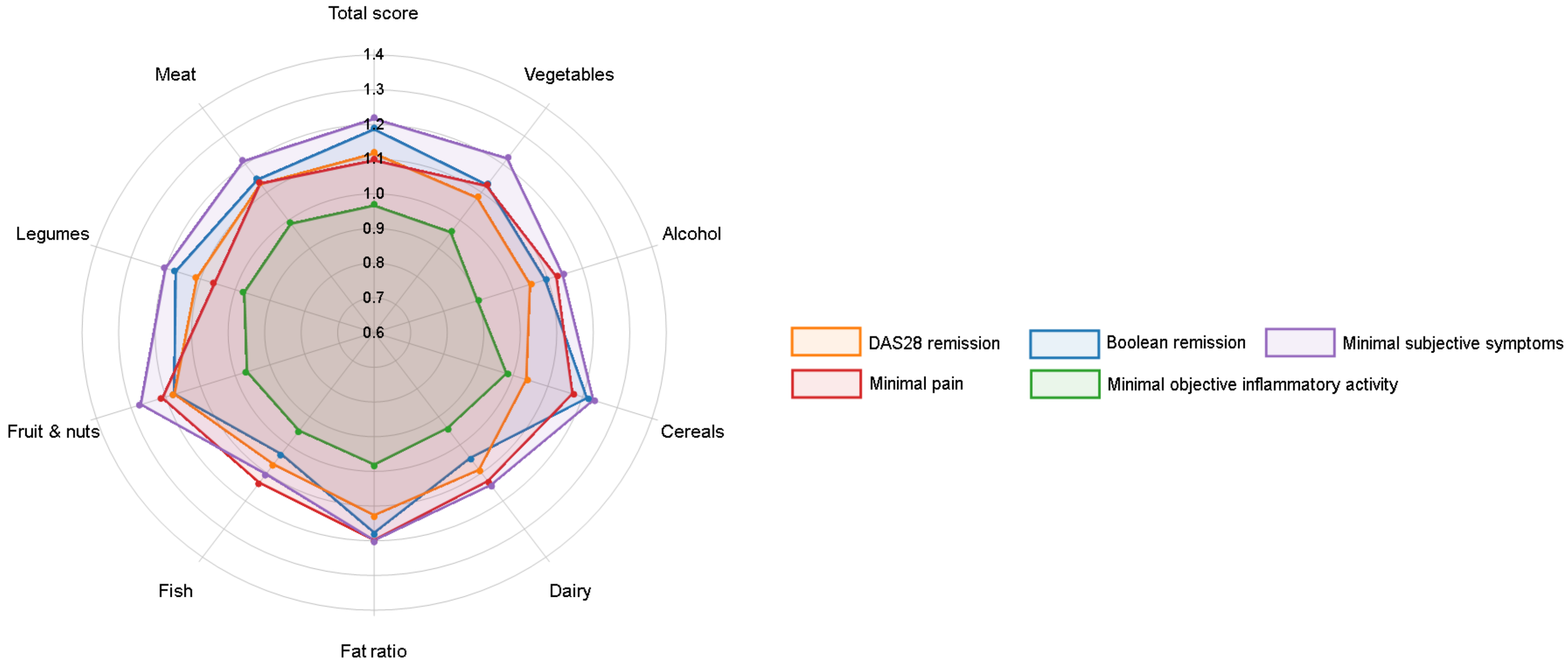

Supplementary Figure 3. Radar plot displaying risk ratios for remission at 6 months post-diagnosis in both seropositive and seronegative RA, based on leave-one-out analyses of Mediterranean diet score components. Analyses were adjusted for age, sex, region, smoking status, physical activity, educational level, body mass index, total energy intake, supplement use, and cortisone use.

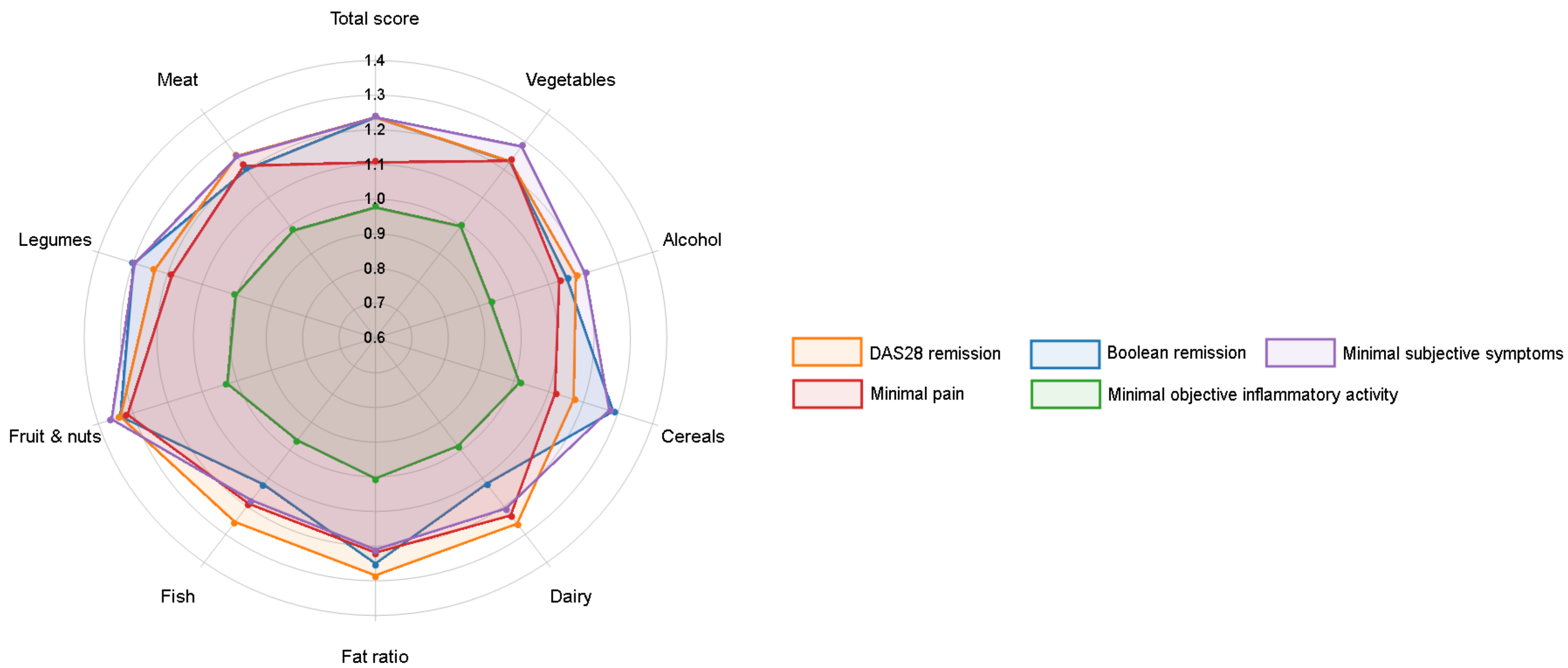

Supplementary Figure 4. Radar plot displaying risk ratios for remission at 6 months post-diagnosis in seropositive RA, based on leave-one-out analyses of Mediterranean diet score components. Analyses were adjusted for age, sex, region, smoking status, physical activity, educational level, body mass index, total energy intake, supplement use, and cortisone use.

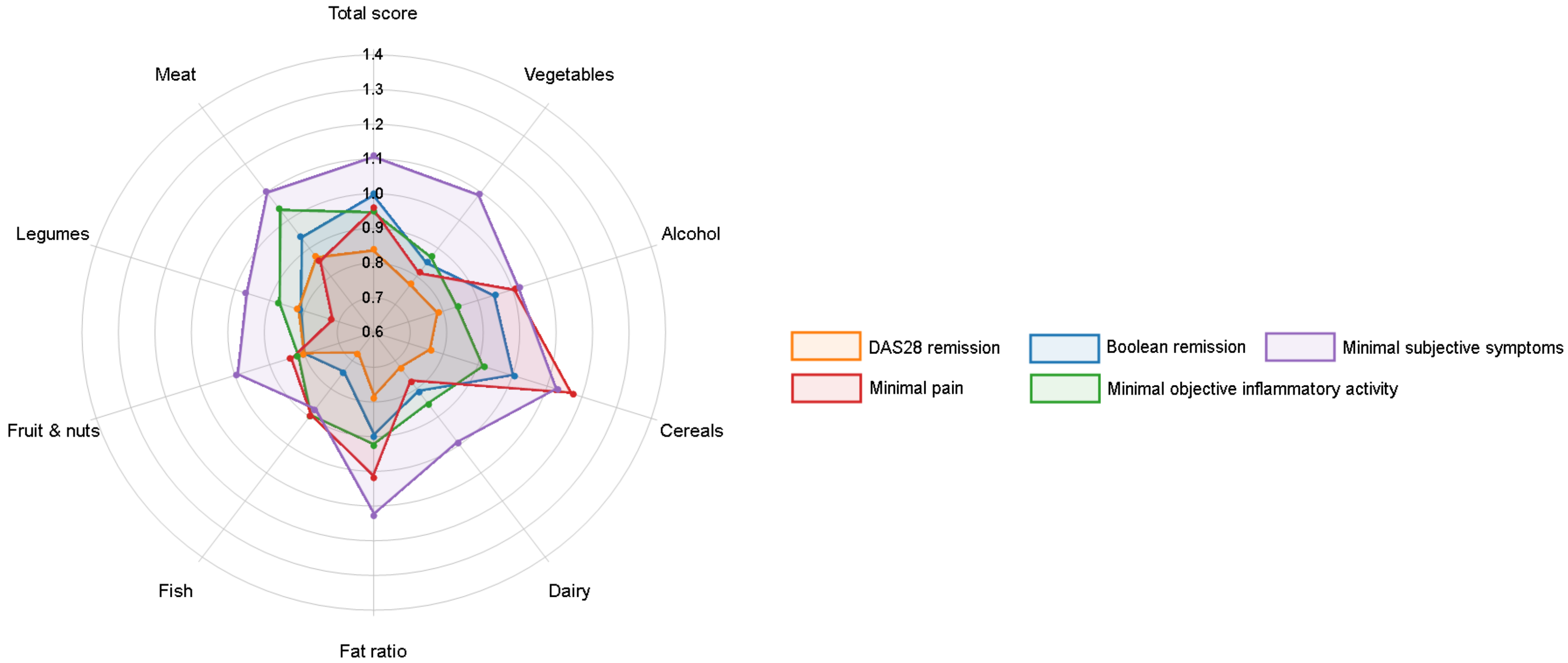

Supplementary Figure 5. Radar plot displaying risk ratios for remission at 6 months post-diagnosis in seronegative RA, based on leave-one-out analyses of Mediterranean diet score components. Analyses were adjusted for age, sex, region, smoking status, physical activity, educational level, body mass index, total energy intake, supplement use, and cortisone use.

Supplementary Table 1. Absolute dietary intakes across Mediterranean diet score tertiles (n=1510)

Dietary intake given as median (IQR) or n (%)	Tertile 1 (0-3) n=604	Tertile 2 (4-5) n=554	Tertile 3 (6-9) n=352
Vegetables, times/day	1.7 (1.0, 2.5)	3.0 (2.2, 4.3)	4.1 (3.0, 5.3)
Fruits and nuts, times/day	0.6 (0.3, 1.1)	1.4 (0.8, 2.1)	2.1 (1.4, 3.1)
Cereals, times/day	2.0 (0.9, 3.0)	2.5 (1.5, 4.1)	3.0 (2.1, 4.2)
Total dairy, times/day	4.1 (2.5, 6.1)	3.2 (2.0, 5.1)	2.5 (1.6, 4.1)
Full-fat dairy, times/day	4.1 (2.0, 5.4)	3.0 (1.4, 4.8)	2.0 (1.0, 3.0)
Legumes, times/week	0.5 (0.5, 0.9)	0.9 (0.5, 1.9)	1.9 (0.9, 3.9)
Fish, times/week	1.9 (1.4, 2.8)	3.2 (1.8, 4.4)	4.4 (3.2, 5.8)
Meat and meat products, times/week	8.2 (4.6, 11.3)	8.2 (5.1, 11.7)	7.3 (4.8, 10.8)
Red meat and meat products, times/week)	6.8 (3.8, 9.9)	6.8 (4.1, 10.3)	5.9 (3.7, 9.2)
Monounsaturated to saturated fatty acid ratio	0.7 (0.6, 0.8)	0.8 (0.7, 0.9)	0.8 (0.8, 0.9)
Moderate alcohol consumption (10–50 g/day for men, 5–25 g/day for women)	96 (16)	188 (34)	204 (58)

Supplementary Table 2. Characteristics of study participants and individuals lost to follow-up

Characteristic given as n (%) or mean (SD)	Study participants n=1510	Lost to follow-up n=680
Age, years	54.9 (13.7)	54.4 (14.5)
Female participants	1066 (71)	492 (72)
Healthcare region		
Stockholm-Gotland	1058 (70)	382 (56)
Southeast	108 (7)	104 (15)
West	31 (2)	40 (6)
South	264 (18)	74 (11)
Uppsala-Örebro	49 (3)	80 (12)
Smoking status		
Current smokers	436 (29)	198 (29)
Former smokers	503 (33)	237 (35)
Physically active	1233 (82)	547 (80)
High education	831 (55)	374 (55)
Autoantibody-positive	1146 (77)	515 (77)
Shared epitope	783 (75)	365 (75)
Body mass index, kg/m^2	25.7 (4.6)	25.8 (4.6)
Mediterranean diet score	4.0 (1.9)	4.0 (1.8)

Missing data (% of total N=1510) were: smoking status 1.1%, physical activity 0.4%, autoantibody status 0.8%, shared epitope 30.9%, and body mass index 0.3%

Supplementary Table 3. Absolute remission at 6 months post-diagnosis, stratified by sex, autoantibody status, and smoking status

	All	Sex		Autoantibody status		Smoking status		
		Female	Male	Positive	Negative	Never smoker	Former smoker	Current smoker
Remission given as n (%)	n=1510	n=1066	n=444	n=1146	n=352	n=555	n=503	n=436
DAS28 remission	752 (52)	496 (49)	256 (60)	563 (52)	182 (53)	268 (51)	253 (52)	223 (54)
Boolean remission	434 (30)	285 (28)	149 (35)	324 (31)	105 (31)	149 (29)	152 (32)	128 (31)
Minimal subjective symptoms	548 (38)	360 (35)	188 (43)	418 (38)	124 (36)	190 (36)	190 (39)	162 (39)
Minimal pain	496 (34)	334 (33)	162 (38)	370 (34)	120 (35)	171 (32)	182 (37)	137 (33)
Minimal objective inflammatory activity	872 (60)	599 (58)	273 (64)	657 (60)	208 (60)	323 (61)	289 (59)	251 (59)

Missing data (% of total N=1510) were: DAS28 remission 4.4%, Boolean remission 5.3%, minimal subjective symptoms 3.6%, minimal pain 3.3%, and minimal objective inflammatory activity 3.6%

Supplementary Table 4. Risk ratios and 95% confidence intervals for remission at 6 months after MTX treatment, across tertiles of Mediterranean diet score (n=1043)

	N total	N remission (%)	RR (95% CI)[1]	RR (95% CI)[2]	RR (95% CI)[3,4]
DAS28 remission					
Tertile 1 (0-3)	399	217 (54)	Reference	Reference	Reference
Tertile 2 (4-5)	371	200 (54)	1.01 (0.88, 1.14)	1.00 (0.88, 1.14)	0.99 (0.87, 1.12)
Tertile 3 (6-9)	231	137 (59)	1.12 (0.97, 1.29)	1.09 (0.95, 1.26)	1.09 (0.95, 1.26)
Boolean remission					
Tertile 1 (0-3)	394	120 (30)	Reference	Reference	Reference
Tertile 2 (4-5)	368	130 (35)	1.17 (0.95, 1.43)	1.14 (0.93, 1.38)	1.14 (0.94, 1.40)
Tertile 3 (6-9)	231	88 (38)	1.29 (1.03, 1.61)	1.23 (0.99, 1.55)	1.24 (0.99, 1.56)
Minimal subjective symptoms					
Tertile 1 (0-3)	403	151 (37)	Reference	Reference	Reference
Tertile 2 (4-5)	372	160 (43)	1.15 (0.97, 1.37)	1.13 (0.96, 1.34)	1.12 (0.95, 1.33)
Tertile 3 (6-9)	235	109 (46)	1.27 (1.05, 1.53)	1.23 (1.02, 1.49)	1.22 (1.01, 1.48)
Minimal pain					
Tertile 1 (0-3)	400	136 (34)	Reference	Reference	Reference
Tertile 2 (4-5)	375	131 (35)	1.02 (0.85, 1.24)	1.04 (0.86, 1.25)	1.00 (0.83, 1.22)
Tertile 3 (6-9)	239	96 (40)	1.19 (0.97, 1.46)	1.18 (0.96, 1.46)	1.10 (0.88, 1.37)
Minimal objective inflammatory activity					
Tertile 1 (0-3)	402	245 (61)	Reference	Reference	Reference
Tertile 2 (4-5)	372	238 (64)	1.07 (0.96, 1.19)	1.06 (0.95, 1.18)	1.06 (0.94, 1.18)
Tertile 3 (6-9)	233	141 (61)	1.02 (0.89, 1.16)	1.01 (0.88, 1.15)	1.01 (0.88, 1.16)

[1]Adjusted for age, sex, and region
[2]Adjusted for age, sex, region, smoking status, physical activity, educational level, body mass index, total energy intake, supplement use, and cortisone use
[3]Adjusted for age, sex, region, smoking status, physical activity, educational level, body mass index, total energy intake, supplement use, cortisone use, baseline 28-swollen joint count, 28-tender joint count, patient global assessment, and C-reactive protein
[4]958 participants were included in this analysis
